# Co-circulation of two genotypes of dengue virus serotype 3 in Guangzhou, China, 2009

**DOI:** 10.1186/1743-422X-9-125

**Published:** 2012-06-22

**Authors:** Tao Jiang, Xue-Dong Yu, Wen-Xin Hong, Wei-Ze Zhou, Man Yu, Yong-Qiang Deng, Shun-Ya Zhu, E-De Qin, Jian Wang, Cheng-Feng Qin, Fu-Chun Zhang

**Affiliations:** 1 State Key Laboratory of Pathogen and Biosecurity, Beijing Institute of Microbiology and Epidemiology, No.20 Dongda Street, Fengtai District, Beijing, 100071, China; 2 Guangzhou No.8 People’s Hospital, Guangzhou Medical College, Guangzhou, 510060, China; 3 The Third People’s Hospital of Huizhou, Huizhou, 516002, China

**Keywords:** Dengue virus type 3, Family cluster, Genotype, Co-circulation

## Abstract

Dengue is emerging as the most important mosquito borne viral disease in the world. In mainland China, sporadic and large outbreaks of dengue illness caused by the four serotypes of dengue virus (DENV-1 to DENV-4) have been well documented. Guangdong province is the major affected area in China, and DENV-1 has dominantly circulated in Guangdong for a long time. In this study, a family cluster of DENV-3 infection in Guangzhou was described. Three cases were diagnosed as dengue fever based on clinical manifestation, serological and RT-PCR assays. Two DENV-3 strains were isolated in C6/36 cells and the complete genome sequences were determined. Phylogenetic analysis revealed that the new DENV-3 isolates from the family cluster were grouped within genotype III. Considering the fact that several DENV-3 strains within genotype V were also identified in Guangzhou in 2009, at least two genotypes of DENV-3 co-circulated in Guangzhou. Careful investigation and virological analysis should be warranted in the future.

## Background

Dengue is increasing in both frequency and magnitude worldwide, posing a heavy public health and economic burden especially in tropical and subtropical countries. Today, dengue ranks as the most important mosquito-borne viral disease in the world. Annually, up to 50 million human infections occur with 22, 000 deaths mainly in children [1]. Even, population growth, urbanization, international travel, and global warming continuously enhance vector transmission and disease outbreaks [2]. Dengue virus (DENV) contains four serotypes, and each of them can cause a wide spectrum of clinical manifestations, including mild dengue fever (DF), severe dengue haemorrhagic fever (DHF) and deadly dengue shock syndrome (DSS). Although intensive efforts have been made for decades, no preventive vaccines or antiviral drugs is currently available. The pathogenesis of DHF and DSS remains poorly understood. However, secondary infection with another DENV serotypes clearly increased the risk of severe diseases via the mechanism of antibody dependent enhancement (ADE) [3-5]. Epidemiological and *in vivo* data also indicated that anti-DENV antibodies mediated pathogenesis of a second heterotypic DENV infection [6-8].

Mainland China has experienced large outbreaks of DF during World War II, after that dengue disappeared for about 30 years. Since 1978, mainland China has seen a resurgence of dengue, epidemics involving hundreds of thousands of people have occurred in many provinces of Southern China, including Hainan, Guangdong, Guangxi, Fujian, Yunnan and Zhejiang provinces [9-14]. Currently, DF is listed as the notifiable infectious disease by the Ministry of Health, China. The recent epidemiology of dengue in China is characterized by a 3–5 year cycle. Most cases are DF, and only a few DHF or DSS cases have been reported over the last decade in mainland China [9,10,13].

In dengue endemic country, the presence of four serotypes of DENV is common, and co-circulation of multiple dengue serotypes in the same area has been well documented [15-17]. Guangdong province has been recognized as the major affected area of China. Although all four serotypes of DENV have been isolated in China, the dominant serotype circulating in Guangdong is DENV-1, no other serotypes has been recorded since 2001 [9,10,13,18]. Large DF outbreaks involving more than 1000 cases caused by DENV-1 have been described in Guangdong, China in 2002 and 2006, respectively [13,19].

In this study, we sought to determine the cause of a family cluster of DF in Guangzhou, Guangdong province, China in 2009, and analyze the possible origin of these emerging isolates responsible for the epidemic.

## Materials and methods

### Case description

On Aug 6, 2009, three adult family members admitted to Guangzhou No.8 People’s Hospital as suspected DF cases. The 30-year-old son firstly had a sudden fever with headache, then his father (56-year-old) and mother (50-year-old) fell ill subsequently in the following two days. All the three cases developed typical DF symptoms, including fever, headache, chills, rash, muscle and joint pain, and anorexia. The couples developed diarrhoea, and none of them showed vomiting. The tourniquet tests were all positive. All patients recovered uneventfully and discharged on Aug 11, 2009.

### Ethics statement

The research was approved by the Review Board of Guangzhou No.8 People’s Hospital and the Ethical Committee of State Key Laboratory of Pathogen and Biosecurity. Informed consent was obtained from patients.

### Serological assay and RT-PCR

Acute phrase sera were subjected to serological assays using IgM and IgG capture ELISA kit (PanBio, Queensland, Australia) according to the manufacturer’s instruction. RT-PCR assays were performed to detect and typing of DENVs as previously described [20].

### Virus isolation and identification

Acute phase sera from the three patients were inoculated in C6/36 mosquito cells (*Aedesalbopitus* clone) and maintained in 1640 medium (Life Technologies, CA, USA) supplement with 2% fetal bovine serum (Life Technologies) at 28 °C in 5% CO_2_. When complete cytopathic effects (CPE) were observed, culture supernatants from positive samples were collected and stored at −70 °C until use. Indirect immunofluscence assay (IFA) was performed as previously described [21].

### Sequencing of complete genome of DENV-3 isolates

The viral RNA was extracted from 200 μl of DENV-3 infected C6/36 culture supernatant using Purelink RNA mini kit (Life Technologies) in accordance with the manufacturer’s instructions. A total of 11 overlapping amplicons spanning the complete genomic region were amplified using 11 pairs of primers. The PCR products were sequenced and assembled. The 5’ and 3’ untranslated regions (UTRs) of viral genome of each isolate were determined using a rapid amplification of either 5’ or 3’ cDNA ends (RACE) kit (Roche, Mannheim, Germany) followed the manufacturer’s recommendation. All primers can be found in Table 1.

**Table 1:** Primers used for sequencing reactions

**Primer Name**	**Location**^***a***^	**Sequence (5'-3')**	**Product (bp)**
F1	1-22	AGTTGTTAGTCTACGTGGACCG	1054
R1	1036-1054	CGTGGGCTTGTTCTTAGCC	
F2	857-877	GCCCATTACATAGGCACTTCC	1199
R2	2036-2055	CTTTCCCCAAAAGGAGGTTC	
F3	1946-1965	GAGGATGGACAAGGGAAAGC	1403
R3	3326-3348	CACCATTCGTGTATCAACTTCCC	
F4	3224-3245	GGAAAATTGGAGCTGGACTTCA	1001
R4	4206-4224	GCCACTAATGGTCCAGCCA	
F5	4112-4131	CTCAAAAGGAGAAGCTGGCC	1208
R5	5299-5319	CGCATTGTGAACGTTGCGTG	
F6	5252-5274	GCAACAAAATCTGAACACACAGG	1071
R6	6301-6321	GAATAAGTGCGGGCATCAAGC	
F7	6059-6077	AAGTCAGCCGCCATAGACG	1389
R7	7428-7447	AGGTGATCCTTCCCAGAGTG	
F8	7032-7049	AGGCAGTGGTCCTGATGG	1143
R8	8153-8174	GCATTCCTCCATGTTTCCTTTG	
F9	8015-8032	TCACCAAGCCCAACAGTG	1528
R9	9520-9542	TGGCCATTCTTTTTAACCTCTCC	
F10	9270-9293	AGCTCACATACCAAAACAAAGTGG	1060
R10	10299-10318	AGCTTCTTCCGTACTGTGGC	
F11^*b*^	10118-10137	GGTCTCACTTCCAGAGCAAC	590
R11	10674-10696	AGAACCTGTTGATTCAACAGCAC	
5'RACE outer primer	661-678	TTGCACGTTCCATAGGTC	
5'RACE inner primer	267-284	CTGCTGTTGGTGGAATGG	

^*a*^ Primer location refers to DENV-3 strain 80–2 [GenBank: AF317645].

^*b*^ Primer F11 was also used as 3’RACE primer.

### Sequence alignment and phylogenetic analysis

The complete nucleotide sequences of the complete sequences of coding region or envelope (E) gene of global DENV-3 strains were retrieved from GenBank (Table 2). Multiple sequence alignment was carried out employing the CLUSTAL W program [22]. Phylogenetic analyses based on the nucleotide sequence of complete coding region of 44 DENV-3 or complete envelope gene of 58 DENV-3 were carried out by Neighbor-Joining method using MEGA version 5.05 or by Bayesian method using BEAST version 1.7.1 [23,24]. The Neighbor-Joining trees were constructed by Tamura-Nei model with gamma-distribution of among-site [25]. The Bayesian trees were inferred by Markov Chain Monte Carlo (MCMC) for 5,000,000 generations, sampling at every 100 the generations. Sequences of the DENV-1 strain WestPac [GenBank: U88535], DENV-2 strain NGC [GenBank: AF038403] and DENV-4 strain B5 [GenBank: AF289029] were used as outgroups.

**Table 2:** DENV-3 isolates investigated in this study

**Isolate**	**Year of isolation**	**Geographical origin**	**GenBank Accession No**.	**Genotype**
GZ1D3	2009	China: Guangdong	In this study	III
GZ2D3	2009	China: Guangdong	In this study	III
09/GZ/11144	2009	China: Guangdong	HM466966 (E)	III
09/GZ/11194	2009	China: Guangdong	HM466967 (E)	III
09/GZ/13105	2009	China: Guangdong	HM466968 (E)	III
09/GZ/10616	2009	China: Guangdong	HM466964 (E)	III
Zhejiang/08/09	2009	China: Zhejiang	GU721065	III
Zhejiang/15/09	2009	China: Zhejiang	GU721066	III
Zhejiang/17/09	2009	China: Zhejiang	GU721067	III
Zhejiang/27/09	2009	China: Zhejiang	GU721068	III
Zhejiang/31/09	2009	China: Zhejiang	GU721069	III
ZJYW2009	2009	China: Zhejiang	JF504679	III
07CHLS001	2007	China: Zhejiang	EU367962	II
DTID-ZJU04	2009	China: Zhejiang	GU189648	II
09/GZ/1081	2009	China: Guangdong	HM466962 (E)	V
09/GZ/1483	2009	China: Guangdong	HM466963 (E)	V
09/GZ/10806	2009	China: Guangdong	HM466965 (E)	V
80-2	1980	China: Guangxi	AF317645	V
ND143	2007	India	FJ644564	III
DEL-72	2008	India	GQ466079	III
DENV-3/LK/BID-V2405	1983	Sri Lanka	GQ199887	III
DENV-3/LK/BID-V2409	1997	Sri Lanka	GQ252674	III
DENV-3/MX/BID-V2989	2007	Mexico	FJ898442	III
DENV-3/US/BID-V2103	2000	USA	FJ547071	III
DENV-3/US/BID-V1080	2006	USA	EU529699	III
DENV-3/US/BID-V1620	2005	USA	FJ182010	III
DENV-3/US/BID-V1090	1998	USA	EU529703	III
DENV-3/US/BID-V1043	2006	USA	EU482555	III
DENV-3/VE/BID-V2179	2000	Venezuela	FJ639750	III
DENV-3/VE/BID-V2481	2007	Venezuela	GQ868586	III
DENV-3/NI/BID-V2419	1998	Nicaragua	GQ199886	III
DENV-3/NI/BID-V2420	1994	Nicaragua	FJ882576	III
DENV-3/NI/BID-V2649	2008	Nicaragua	FJ873813	III
DENV-3/NI/BID-V4761	2009	Nicaragua	HM181972	III
DENV-3/NI/BID-V3055	2008	Nicaragua	GQ199860	III
DENV-3/NI/BID-V5498	2010	Nicaragua	JF937633	III
DENV-3/LC/BID-V3929	2001	Saint Lucia	GQ868616	III
DENV-3/BR/BID-V2400	2007	Brazil	FJ850092	III
DENV-3/BR/BID-V2977	2001	Brazil	FJ898446	III
DENV-3/BR/BID-V3606	2007	Brazil	GU131876	III
DENV-3/IPC/BID-V3832	2007	Cambodia	GU131917	II
DENV-3/KH/BID-V2082	2003	Cambodia	FJ639725	II
DENV-3/IPC/BID-V3820	2006	Cambodia	GU131908	II
DENV-3/IPC/BID-V4286	2007	Cambodia	GU131937	II
DENV-3/KH/BID-V2080	2003	Cambodia	FJ639723	II
DENV-3/VN/BID-V1013	2006	Viet Nam	EU482457	II
DENV-3/VN/BID-V1911	2008	Viet Nam	FJ547066	II
C0360/94	1994	Thailand	AY923865	II
C0331/94	1994	Thailand	AY876494	II
DENV-3/TH/BID-V2315	2001	Thailand	FJ744729	II
DENV-3/TH/BID-V3360	1973	Thailand	GQ868593	II
PF90/3050	1990	French Polynesia	AY744679	I
PF92/4190	1992	French Polynesia	AY744684	I
den3-88	1988	Indonesia	AY858038	I
FW01	2004	Indonesia	AY858040	I
H87	1956	Philippines	M93130	V
BS-PRico63	1963	Puerto Rico	AY146762	IV
1339	1977	Puerto Rico	AY146761	IV

## Results

All three family members were diagnosed as DF according to the new guideline of World Health Organization [26]. Laboratory tests disclosed low WBC and lymphocytes counts for all the three cases. Normal platelet counts were recorded for two cases, while that of the mother was low. None of the patients presented plasma leakage, severe bleeding, or severe organ involvement. All cases recovered in a week post admission.

The acute phase sera from all the three family members were positive for dengue IgM antibody, but negative for IgG antibody. Two of the three cases were positive for DENV-specific RT-PCR. DNA sequencing of the PCR products and blast analysis revealed closely homologous with DENV-3. Considering the fact that DENV-3 has not been described in Guangdong for many years, all the three samples were inoculated into C6/36 cells to isolate the viruses. Typical CPE were observed six or seven days post inoculation for two of three samples. After another passage in C6/36 cells, two strains were isolated and named with GZ1D3 and GZ2D3, respectively. Both strains were further confirmed by IFA using dengue specific monoclonal antibody.

**Figure 1:**
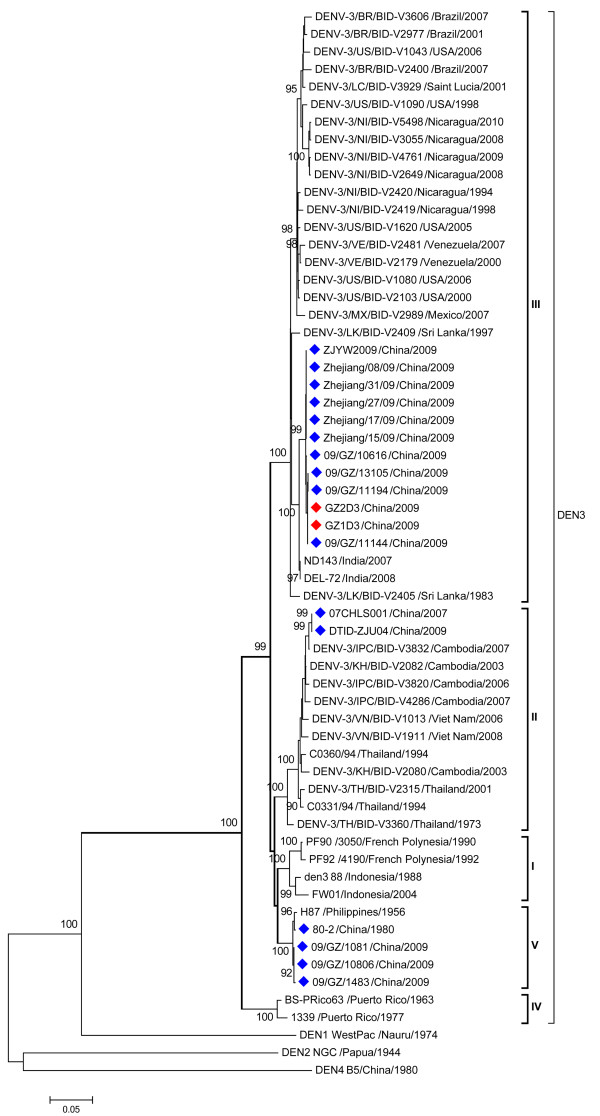
**Phylogenetic tree based on the complete envelope gene from 58 DENV-3 strains sampled globally.** The evolutionary history was inferred using the neighbor-joining method with MEGA 5 software [24]. Each strain is abbreviated with strain name and country of origin followed by the year of isolation. Bootstrap values greater than 0.9 based on 1000 replicates are shown for key nodes. The tree was rooted using DENV-1 strain Nauru, DENV-2 strain New Guinea C and DENV-4 strain B5 as outgroups. The newly DENV-3 isolates in the study are marked with red squares and other Chinese DENV-3 isolates taken for comparison are marked with blue squares.

**Figure 2:**
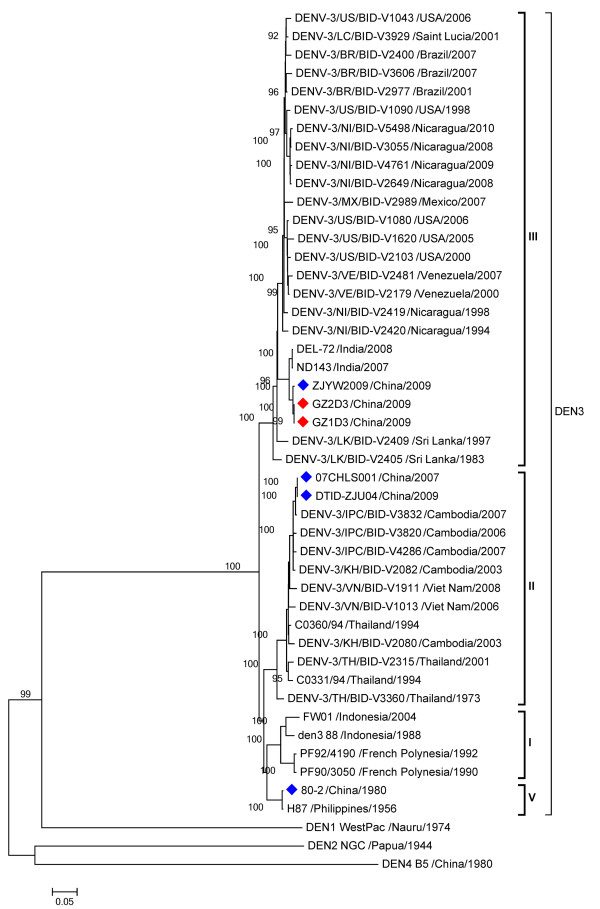
**Phylogenetic tree based on complete sequences of coding region of viral genome from 44 DENV-3 strains sampled globally.** The evolutionary history was inferred using the neighbor-joining method with MEGA 5 software [24]. Bootstrap values greater than 0.9 based on 1000 replicates are shown for key nodes. The tree was rooted using DENV-1 strain Nauru, DENV-2 strain New Guinea C and DENV-4 strain B5 as outgroups. The newly DENV-3 isolates in the study are marked with red squares and other Chinese DENV-3 isolates taken for comparison are marked with blue squares.

Finally, the complete genome sequences of the isolates were determined, assembled and submitted to GenBank [GenBank: GU363549; JN662391]. Both strains were highly homologous (99.9%) with only three nucleotide differences. Phylogenetic analysis based on the complete envelope gene classified DENV-3 isolates into five genotypes (Figure 1), which was confirmed by the Bayesian method (Additional file 1: Figure S1). Phylogenetic tree based on the complete sequence of coding region of DENV-3 genome showed the same genotype classification (Figure 2). The newly isolated DENV-3 strains belong to the genotype III, clustering with other DENV-3 isolates circulating in China in 2009 and in India in 2007 and 2008 (Figure 1). Interestingly, three additional DENV-3 strains isolated in Guangzhou in 2009 (09/GZ/1081, 09GZ/1483 and 09/GZ/10806) [27] belong to genotype V (Figure 1 & Additional file 1: Figure S1), which indicated that two genotypes of DENV-3 were co-circulating in Guangdong, 2009.

## Discussion

In the present study, a family cluster of DENV-3 infections in Guangzhou, China was described. Three family members were diagnosed as DF, and all recovered finally. All the three family members recalled mosquito biting before illness, and none of them went aboard or on trip recently. Although family cluster of vector-borne diseases have been intensively described [28,29], information regarding DF family cluster is limited. There is no doubt that any cluster of cases is of great concern and should be thoroughly investigated. Dengue can cause both the large epidemics and sporadic infections. The recognition of clustering of disease is important for medical providers and public health personnel in treating and controlling the disease, because multiple infections can occur simultaneously or following an index case. In this study, strict mosquito control measures were initiated immediately after confirmation of the DF cases, and no further cases were reported nearby thereafter.

Whether dengue is endemic in Guangdong remains disputable. Most dengue epidemics in Guangdong were initiated by the introduction of virus from imported cases [13,18,19,27,30], however, in this study none of the family member travelled aboard. Further epidemiology investigation also did not identify imported case nearby either. The origin of these DENV-3 isolate is really interesting. Since DENV-1 has circulated in Guangzhou for about ten years, the new DENV-3 has potential to increase the rate of secondary heterotypic infection. Furthermore, the previous studies showed that epidemic DHF has appeared in association with DENV-3 [31-33]. In the Americas, DENV-3 presented greater epidemic potential and virulence [20]. Whatever, the emerging DENV-3 in Guangzhou might represent a risk factor for severe dengue illness, careful investigation and surveillance should be warranted in the future.

Most importantly, phylogenetic analysis demonstrated that at least two different genotypes of DENV-3 were co-circulated in Guangdong, China in 2009, which partly agree with the findings of the previous study [27]. Five genotypes of DENV-3 have been reported [20,34]. The newly isolates in Guangdong form a distinct cluster with other Chinese isolates sampled from Zhejiang province in 2009 [35]. All these genotype III DENV-3 strains were closely related to those sampled in India in 2007 and 2008, suggesting these Chinese isolates might be imported from India. Previously, the introduction of new genotype III of DENV-3 has been recognized as one of the factors leading to the emergence of DHF in Pakistan and India [33,36]. These strains are therefore interesting and their virological characterization and virulence analyses are currently underway.

However, three additional DENV-3 strains (09/GZ/1081, 09GZ/1483, and 09/GZ/10806), belonging to genotype V, were also identified in Guangzhou, 2009 in a separate study [27]. In addition, two strains (07CSHL001 and DTID-ZJU04) sampled from China, were grouped within genotype II. The origin of these isolates is difficult to determine without further information. The situation that multiple genotypes of DENV-3 co-circulated in Guangzhou, China deserves close concern and careful investigation.

## Competing interests

The authors declare that they have no competing interests.

## Authors’ contributions

TJ, XDY, CFQ and FCZ: designed the study, did laboratory testing, analyzed the test results. TJ, EDQ and CFQ co-wrote and edited the manuscript. WXH and WZZ participated in the gene sequencing and phylogenetic analysis. MY, YQD, SYZ, EDQ and JW took samples and did laboratory testing and virus isolation. All authors read and approved the final manuscript.

## Supplementary Material

Additional file 1 **Figure S1.** Phylogenetic tree based on the complete envelope gene from 58 DENV-3 strains by Bayesian method. The evolutionary history was inferred using BEAST 1.7.1 software. The tree was rooted using DENV-1 strain Nauru, DENV-2 strain New Guinea C and DENV-4 strain B5 as outgroups. The newly described DENV-3 isolates in the study are marked with red squares and other Chinese DENV-3 isolates taken for comparison are marked with blue squares. (TIFF 1759 kb) Click here for file
