# Supplementary figures and images for: Co-circulation of two genotypes of dengue virus serotype 3 in Guangzhou, China, 2009
Source: Virol J. 2012 Jun 22;9:125. doi: 10.1186/1743-422X-9-125 (PMC3463466; doi:10.1186/1743-422X-9-125)

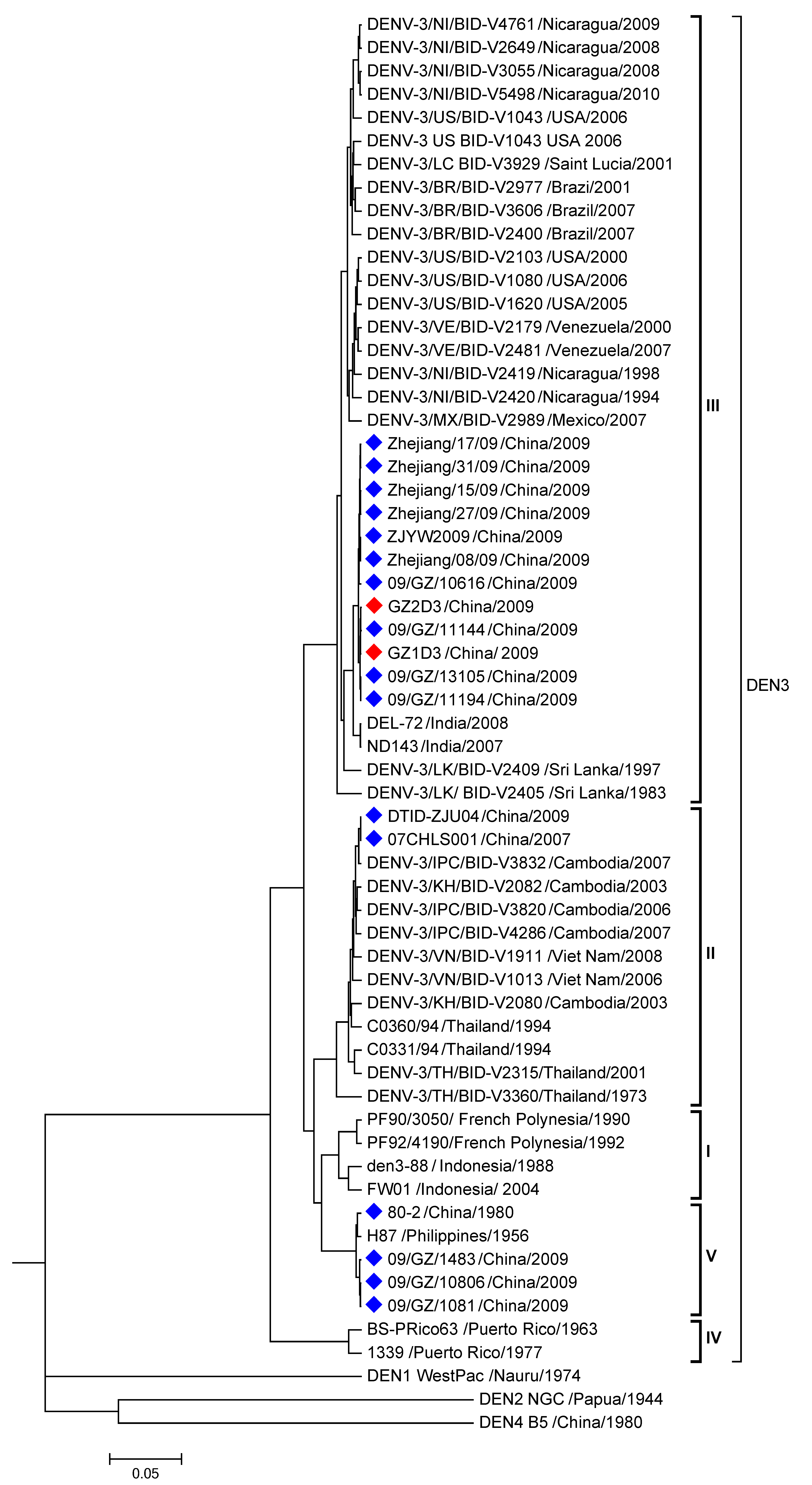

Supplement: Additional file 1 — Figure S1. Phylogenetic tree based on the complete envelope gene from 58 DENV-3 strains by Bayesian method. The evolutionary history was inferred using BEAST 1.7.1 software. The tree was rooted using DENV-1 strain Nauru, DENV-2 strain New Guinea C and DENV-4 strain B5 as outgroups. The newly described DENV-3 isolates in the study are marked with red squares and other Chinese DENV-3 isolates taken for comparison are marked with blue squares. (TIFF 1759 kb) [file 1743-422X-9-125-S1.tiff]
